# Clustering of genes into regulons using integrated modeling-COGRIM

**DOI:** 10.1186/gb-2007-8-1-r4

**Published:** 2007-01-04

**Authors:** Guang Chen, Shane T Jensen, Christian J Stoeckert

**Affiliations:** 1 Department of Bioengineering, University of Pennsylvania, 240 Skirkanich Hall, 3320 Smith Walk, Philadelphia, Pennsylvania 19104, USA; 2 Center for Bioinformatics, University of Pennsylvania,1420 Blockley Hall, 423 Guardian Drive, Philadelphia, Pennsylvania 19104, USA; 3 Department of Statistics, The Wharton School, University of Pennsylvania, 463 Jon M. Huntsman Hall, 3730 Walnut Street, Philadelphia, Pennsylvania 19104, USA; 4 Department of Genetics, School of Medicine, University of Pennsylvania, 415 Curie Boulevard, Philadelphia, Pennsylvania 19104, USA

## Abstract

We present a Bayesian hierarchical model and Gibbs Sampling implementation that integrates gene expression, ChIP binding, and transcription factor motif data in a principled and robust fashion. COGRIM was applied to both unicellular and mammalian organisms under different scenarios of available data. In these applications, we demonstrate the ability to predict gene-transcription factor interactions with reduced numbers of false-positive findings and to make predictions beyond what is obtained when single types of data are considered.

## Abstract 2

COGRIM, an implementation that integrates gene expression, ChIP binding and transcription factor motif data, is described and applied to both unicellular and mammalian organisms.

## Background

The interactions of transcriptional regulators of gene expression with each other and their target genes are often summarized in the form of regulatory modules and networks, which can be used as a basis for understanding cellular processes. The computational procedures that are employed to identify gene regulatory modules and networks have traditionally used information from expression data, binding motifs, or genome-wide location analysis of DNA-binding regulators [1]. A typical approach has been to first use clustering algorithms on expression data to find sets of co-expressed and potentially co-regulated genes, and then the upstream regulatory regions of the genes in each cluster are analyzed for common cis-regulatory elements (motifs) or modules of several cis-regulatory elements located in close proximity to each other [2]. These cis-regulatory elements are the potential binding sites of transcription factor (TF) proteins, which bind directly to the DNA sequence in order to increase or decrease transcription of specific target genes. This computational strategy can also be employed using chromatin immunoprecipitation (ChIP) technology, which identifies genomic sequences that are enriched for physical binding of a particular TF [3]. Although such approaches have proven to be useful, their power is inherently limited by the fact that each data source provides only partial information: expression data provides only indirect evidence of regulation, upstream regulatory region searches provide only potential binding sites that may not be bound by TFs, and ChIP binding data provides only physical binding information that may not be functional in terms of controlling gene expression.

There has been substantial recent research into the integration of biological data sources for the discovery of regulatory networks. Different approaches taken have included heuristic algorithms [4,5], linear models [6-12], and probabilistic models [13,14]. The GRAM algorithm [4] employed exhaustive search and arbitrary parameter thresholds on ChIP binding and expression data to discover regulatory networks in *Saccharomyces cerevisiae*. ReMoDiscovery [5] was developed to combine all three data types - ChIP binding, expression, and TF motif data - but the technique is heuristic with arbitrary parameter thresholds and little systematic modeling. Multivariate regression analysis was presented by Bussemaker and coworkers [7] to infer regulator networks from expression and ChIP binding data, but their model required a stringent binding *P *value threshold. In a 'network component analysis' approach [10-12], ChIP binding data are used to form a connectivity network between genes and TFs, but the network is assumed to be known without error. Based on the assumption that the expression levels of regulated genes depend on the expression levels of regulators, Segal and coworkers [13,14] constructed a probabilistic model that used binding motif features and expression data to identify modules of co-regulated genes and their regulators. This probabilistic model reflected nonlinear properties but required prior clustering of the expression data.

Although these approaches have achieved a certain degree of integration, they have been limited in model extensibility and require *a priori *knowledge of the contribution of each data source in the form of TF binding sites, gene expression clusters, and/or ChIP binding *P *values. We have developed a novel Bayesian hierarchical approach that extends previous linear models [6,7,10] to provide a flexible statistical framework for incorporating different data sources. Building upon this linear model foundation, our extended probabilistic approach achieves a principled balance for the contributions of each data source to the modeling process without requiring predetermined thresholds or clusters. In addition, our model allows us to estimate synergistic and antagonistic interactions between TFs and permits genes to belong to multiple regulons [15], which allows us to model multiple biologic pathways simultaneously.

## Results

### Application to *Saccharomyces cerevisiae*

The model was applied to genome-wide ChIP binding data [3] and approximately 500 expression experiments on *S. cerevisiae *(Additional data file 1 [Supplementary Table 1]). From 106 TFs measured by Lee and coworkers [3], 39 were selected as our validation set, which includes most cell cycle related TFs and some stress response related factors. We used our full estimated regulation matrix *C *to classify target genes for each of our 39 TFs by applying a posterior probability cutoff of 0.5 on each *C*_*ij*_. The 39 TFs and 1542 classified target genes were used to construct a functional yeast transcriptional regulatory network consisting of 2,298 TF and gene interactions (for regulatory networks, see Additional file 1 [Supplementary Figure 1]).

### Classification of target genes by COGRIM versus ChIP binding data alone

For each TF, our model integrates both binding and gene expression data to identify regulated C+ and unregulated C- genes, based on our estimated indicator matrix *C*. Similarly, for each TF, there are two gene sets classified by the binding *P *value from ChIP-ChIP experiments by Lee and coworkers [3]. The set B+ includes genes that appear regulated by the TF based only on ChIP binding data (genes with binding *P *< 0.001). The remaining set B- includes nonregulated genes according to ChIP binding data alone. Combining these two classification sets gives us four different categories for each TF: genes identified to be TF targets in both our model and binding data alone (B+/C+); genes identified to be targets by our model but not the binding data alone (B-/C+); genes predicted as targets by binding data alone but not our model (B+/C-); and, finally, the least interesting set of genes, which are not targets based on either method (B-/C-).

Table 1 gives the number of genes in each group for each of the 39 TFs we examined. Overall, 51% of predicted regulated genes by binding data alone are also identified as regulated by our model (B+/C+). In addition, our method identified an additional 14% of probable target genes (B-/C+) that were not considered by binding data alone using a stringent *P *value threshold (*P *< 0.001).

### MIPS functional category analysis

**Figure 1:**
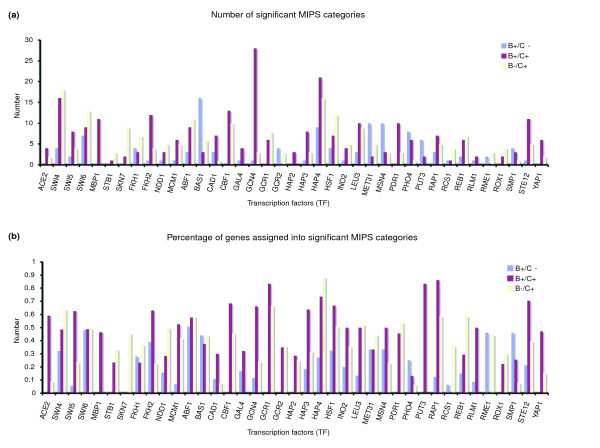
Enrichment of MIPS functional annotations. The hypergeometric distribution was used to calculate *P *values to determine the enrichment of MIPS functional categories, and *P *values smaller than 0.001 were considered to indicate significant over-represention. For each of the 39 TFs analyzed, **(a) **the number of significantly over-represented MIPS categories in the functional targets (B+/C+ [red] and B-/C+ [yellow] clusters) and nonfunctional targets (B+/C- cluster [blue]) are summarized. **(b) **The percentage of genes categorized into significantly over-represented MIPS categories in B+/C+ (red) and B-/C+(yellow) clusters and B+/C- set (blue). TF, transcription factor.

We used the MIPS database [16] to assign a functional category to each gene in our dataset, and tabulated the over-represented functional categories in the set of target genes for each TF. In Figure 1a, we see that for most TFs there was a higher number of significantly over-represented MIPS functional categories for our predicted target genes (B+/C+ and B-/C+ sets) than for the set of target genes predicted by binding data alone but not our model (B+/C-). This same trend is observed when we examine the percentage of genes with significant MIPs categories (Figure 1b). This result validates the assertion that genes found to be regulated in our model, which integrates expression and binding data, are more likely to be functionally related than genes classified by binding data alone.

**Figure 2:**
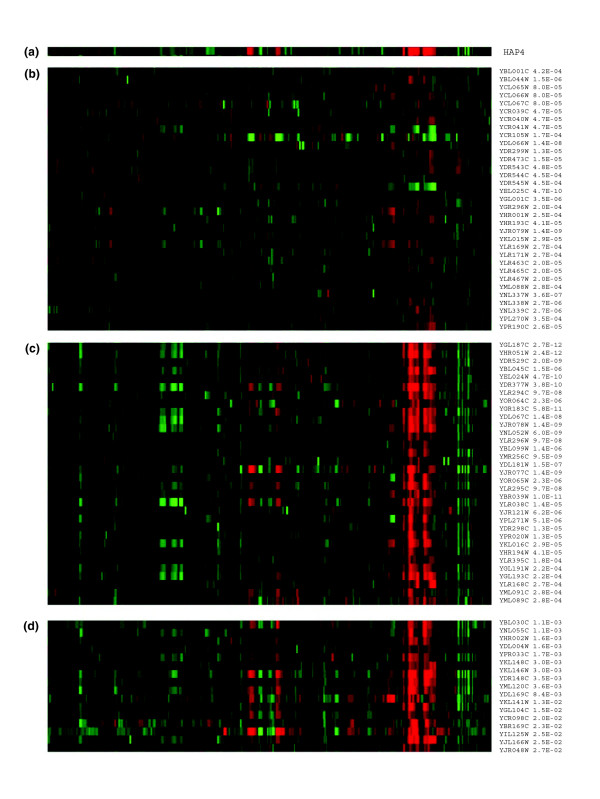
COGRIM improves gene classification in HAP4 case. For each of HAP4 gene clusters, genes are ordered by the ChIP binding *P *value obtained from Lee and coworkers [3]. **(a) **The expression profile of HAP4, a well characterized factor that is involved in respiration, across approximately 500 experiments. **(b) **The B+/C- gene cluster (33 genes). With ChIP binding data alone, these genes are considered HAP4 targets but they do not share similar expression patterns (averaged centered pearson correlation is only 0.06) and none of them was assigned to the MIPS respiration category. COGRIM does not consider these genes as HAP4 functional targets. **(c) **The B+/C+ gene cluster (34 genes). This gene cluster shows high expression correlation (the averaged centered pearson correlation is 0.56), and 23 out of 34 genes were assigned to the MIPS respiration category. **(d) **The B-/C+ gene set (17 genes). These 17 genes were not identified as HAP4 targets by using binding data alone (with *P *value threshold 0.001) but were predicted by COGRIM to be functional targets. They exhibit coherent expression (the averaged centered pearson correlation is 0.60) and nine of them (ybl030c, ydl004w, yfr033c, yjl166w, yjr048w, ykl141w, ykl148c, yml120c, and ynl055c) are involved in respiration. ChIP, chromatin immunoprecipitation.

More detailed analysis also suggests that the functions of genes predicted as regulated by our method are consistent with the known regulatory roles of TFs. For instance, HAP4 is a well characterized factor that is involved in respiration. None of the 33 B+/C- genes considered as HAP4 targets by binding data alone but not by our method were categorized into MIPS respiration, whereas 9 out of 17 B-/C+ genes predicted by our method to be HAP4 targets (but not by binding data alone) were categorized as respiration genes. These nine genes would not be considered as HAP4 targets based on binding data alone with a stringent binding *P *value threshold [3,7]. Not surprisingly, a large portion (23 of the 34) of the B+/C+ genes, which are predicted as regulatory targets by both methods, are categorized as respiration genes. Figure 2 shows the expression patterns of genes in each of these three sets, and it can be clearly seen that the patterns for the genes predicted as functional targets by our method (B+/C+ and B-/C+) are more coherent than the patterns for the genes predicted as targets by binding data alone but not our method (B+/C-). These results indicate that our method has been more effective at predicting regulated genes for HAP4.

### Response to transcription factor deletion experiments

We also analyzed the gene expression response among our three gene sets for the TF deletion experiments from the Rosetta Yeast Compendium [17]. Table 2 shows the change in expression between knockout and wild-type examined within each gene set (B+/C+, B-/C+, B+/C-) for four TFs that have been subjected to deletion experiments and for which expression and ChIP binding data are available. Negative mean values indicate that target genes were downregulated because of TF deletion, which implies that the TF functions as an activator. Based on standard *t*-tests, genes predicted as functionally regulated by our model (B+/C+ and B-/C+) exhibit a significant change in mRNA expression, whereas the response of genes that are classified as regulated by binding data alone but not our method (B+/C-) did not exhibit a significant difference, indicating that our model identified more appropriate TF targets.

### Identifying significant transcription factor interactions

**Figure 3:**
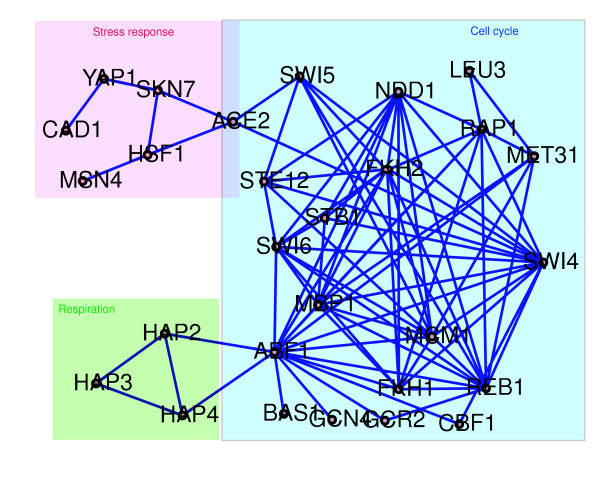
Significant TF pair interactions. Eighty-four TF pairs were identified to have significant synergistic effects on expression of target genes. Nodes represent TFs and edges indicate that two connected TFs form a module to regulate a set of genes. The TF pair is determined to be significant if they share at least four functional target genes and if the posterior interval for the interaction effect term *g*_*jk *_is significantly different from zero (details given in Additional data file 1 [Supplementary methods]). The target genes of each regulator are not shown. Regulators without significant interaction with other TFs are not shown. This network is illustrated with Cytoscape [33]. TF, transcription factor.

Our model was also used to identify 84 TF pairs as having significant interactions, based on shared target genes and a posterior interval for *g*_*jk*_, which was significantly different from zero (for details, see Additional data file 1 [Supplementary methods]). A subset of these paired interactions are shown in Figure 3. Most of the TFs (ACE2, SWI4, SWI5, SWI6, MBP1, FKH1, FKH2, NDD1 and MCM1) connected on the right side of Figure 3 are known cell cycle TFs, whereas the TFs connected in the upper left corner are known to be involved in stress response, and the lower left HAP2-HAP3-HAP4 module regulates respiratory gene expression. Many of these regulatory module relationships are experimentally confirmed (Additional data file 1 [Supplementary Table 2]). For example, MCM1 and FKH2 form a regulatory module to control the expression of cell cycle gene cluster CLB2 [18]. SKN7 was reported to interact with HSF1 and is required for the induction of heat shock genes by oxidative stress [19]. Besides the known SKN7-HSF1 module, we also identified ACE2-HSF1 and ACE2-SKN7 interactions; this supports speculation from previous studies [20-22] that ACE2 may be a co-activator of HSF1 and SKN7, which influences full induction of a subset of the HSF1 and SKN7 target genes.

### Application to serum response factor

Currently, ChIP-chip experiments have only been performed on certain TFs in higher organisms because of limited availability of promoter chips and antibodies. However, in many cases TF binding site predictions from a position weight matrix (PWM) scanning procedure can provide some useful information about potential gene targets, although it is well accepted that ChIP-chip data are generally more reliable. We demonstrate that our COGRIM model can effectively integrate TF binding site data with expression data for target gene prediction in the absence of ChIP binding data by applying our model to serum response factor (SRF), which has a well conserved binding PWM-CArG box [23] and primarily controls expression of muscle and growth factor associated genes. PWM-based sequence scanning data for SRF [24,25] was used to construct prior probabilities for each gene in our dataset (for details, see Additional data file 1 [Supplementary Methods]). We used publicly available gene expression data from the studies of Balza and Misra [26] and Selvaraj and Prywes [27].

Our COGRIM model based on the integration of SRF expression and PWM scan data resulted in 64 predicted SRF gene targets (Additional data file 1 [Supplementary Table 3]). These 64 predicted genes contain 50 that are experimentally validated targets [25], which leaves 14 targets (21.9%) as possible false positives. Using binding site data alone, Sun and coworkers [25] reported a 32.5% false positive rate, which is substantially higher than that with our integrated method. Our predictions also have a low false negative rate, because only three experimentally validated SRF targets were missed. Thus, our COGRIM approach has resulted in target gene predictions with a reduced false positive rate while maintaining a low false negative rate.

**Figure 4:**
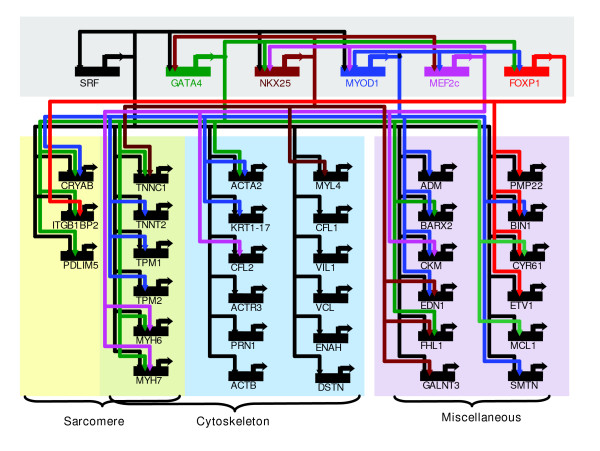
SRF regulatory circuits. Five known SRF co-factors are selected to study their modular regulatory roles. Based on shared target genes and significant interaction effects γ from the model, SRF regulatory circuits are identified as having significant effects on expression of target genes. SRF, serum response factor.

The expression profiles of SRF targets are found to be highly correlated with the SRF probe (average Pearson correlation of 0.62), which again supports the assumption that TF expression can serve as a reasonable proxy for TF regulatory activity. We also examined our predictions in the context of several selected SRF cofactors. The SRF-cofactor regulatory circuits (Figure 4) identified by our COGRIM are consistent with current knowledge of SRF's modular regulatory role [23,26,27]. For example, SRF is known to associate physically with the TF Nkx2.5 and GATA4 to activate the cardiac α-actin and atrial natruretic factor genes [23]. COGRIM also recognized that SRF is the central component of a hierarchical cascade model of muscle-specific gene transcriptional network, and in which SRF both directly and indirectly regulates the expression of genes required for contractile apparatus assembly [25].

### Application to C/EBP-β enhancer

CCAAT/enhancer-binding protein (C/EBP)-β is a basic leucine zipper TF with an important signaling role in the physiology of growth and cancer. We applied COGRIM to identify C/EBP-β target genes using all three available data sources: ChIP binding data, TF binding data from PWM scanning, and gene expression data [28]. The ChIP binding probabilities were calculated from published *P *values [28], whereas the TF binding site probabilities were computed using TESS [24]. Details are contained in Additional data file 1 (Supplementary Methods). Our COGRIM model identified 14 out of 16 experimentally validated C/EBP-β targets [28] and predicted an additional 18 potential target genes. We examined in detail the fold changes of these additional predicted genes, and we found that COGRIM is able to select genes with balanced fold changes between binding and expression data as C/EBP-β targets (Additional data file 1 [Supplementary Table 4]), whereas some of these targets were excluded in previous approaches as a result of applying arbitrary cutoffs in orthogonal analysis [28].

**Figure 5:**
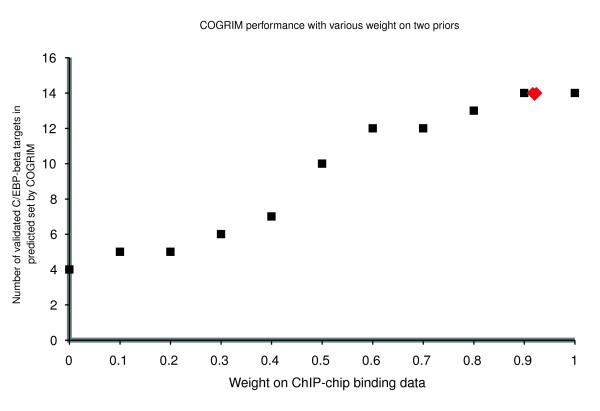
Prediction performance with various weights on two priors. To examine the effect of our prior information on prediction, we used a restricted COGRIM model that assigned fixed weights *w *(ranging from 0 to 1) to the ChIP binding data. The x-axis represents the assigned weights and the y-axis represents the number of predicted true C/EBP-β targets in 16 validated ones (black square spots). The sampling procedure automatically assigned an appropriate weight 0.92 (variance 0.006) to ChIP-chip binding data (red diamond spot). C-EBP, CCAAT/enhancer-binding protein; ChIP, chromatin immunoprecipitation.

Compared with predictions based on single data resource alone, the number of predictions from COGRIM is substantially smaller than the 72 potential targets based on expression data alone or 779 potential targets based on ChIP-chip binding data alone [28], which suggests that our model leads to a substantial reduction in the number of false positives. As illustrated in previous studies [28,29], the use of PWM scanning to identify C/EBP-β regulatory elements has low discriminative power because of substantial variation in the optimal C/EBP binding motif. As a result, C/EBP-β binding site data alone can be used for detection of target genes but leads to an unreasonable level of false positives. This phenomenon is captured in our COGRIM model by the weight variable *w*, which balances the relative quality of the ChIP binding data versus the TF binding site data. For the C/EBP-β application, our model estimated a weight of *w *= 0.92 for the ChIP binding data, which confirms that the TF binding site data are useful in some instances but generally have much less discriminative power than do ChIP binding data. To further examine the effect of our prior information on prediction, we used a restricted COGRIM model that assigned fixed weights *w *(ranging from 0 to 1) to the ChIP binding data. In Figure 5, we see that target gene prediction becomes more precise with increased weight on ChIP-chip binding data, and we also see that our full COGRIM model estimates a weight *w *that is nearly optimal (as measured by prediction of experimentally verified targets).

Moreover, to understand the contribution from expression data, we designed COGRIM to update the indicator *C*_*ij *_without the ChIP binding and motif priors (Additional data file 1 [Supplementary Methods, section 3]). We conducted this designed study with the same expression data on this C/EBP-β case, and identified only 5 out of 15 targets that were experimentally validated (Additional data file 1 [Supplementary Table 5]). As reported above, the full COGRIM, which integrates all three data types, can identify 14 out of 15 validated C/EBP-β targets. Based on this, we may suggest that the expression only contributed about 35% to the predication and ChIP binding data actually contribute much. This better performance of integrative approaches compared with expression data alone is consistent with previous reports [3,14,28]. This application demonstrates the flexibility of our model to integrate several data types (ChIP binding, TF binding sites from PWM scanning and gene expression) simultaneously for the identification of target genes, as well as the ability to achieve an appropriate balance between these different data resources.

### Comparison with previous approaches

Although direct comparison with previous methods is complicated by the diversity of models and limited availability of software, we were able to evaluate our COGRIM model relative to several previous procedures: two heuristic methods (ReMoDiscovery [5] and GRAM [4]), a multiple regression method (MA-Networker) [7], and the linear model without interaction terms (named Model I [Eqn 1] in Materials and methods, below).

Using our yeast application, we compared the predicted gene regulons obtained by each procedure by calculating the within-regulon expression correlation as well as the within-regulon MIPS category enrichment. Both of these measures are averages across the regulons for all 39 TFs examined in detail in our yeast application. Default parameter settings were used for the previous procedures ReMoDiscovery, GRAM, and MA-Networker. As shown in Table 3, COGRIM shows superior average MIPS category enrichment (0.45) and the average correlation of expression (0.37) compared with Model I and the other three methods. The set of genes (B-/C+) predicted by COGRIM but not ChIP binding data alone share similar MIPS and expression measures to the core regulons (B+/C+) predicted by both COGRIM and ChIP binding data alone, which suggests that the 14% additional TF targets predicted by COGRIM are likely to be functional.

We also compared our COGRIM results with Model I and the three previous methods using the Rosetta Yeast Compendium [17] data on gene expression response to TF deletion. For the four TF deletion experiments for which expression and ChIP binding data are also available, we observe lower P values for differential expression from the predicted COGRIM regulons compared with the regulons predicted by Model I and the other methods (Table 4). The superior expression response to TF deletion shown by our COGRIM predicted gene regulons again suggests that our results are more functionally relevant than the results from previous methods. The *P *values obtained by MA-Networker [7] are also generally small, which suggests that this method is also effective at identifying appropriate regulons, although the results from MA-Networker are inferior to COGRIM on the MIPS and expression correlation measures (Table 3).

We suspect that COGRIM's superior performance is, in part, because we include a probabilistic model for each data source, which addresses the inherent uncertainty within each data type, and consider the TF interactions. In contrast, the multiple regression method (MA-Networker) applies an arbitrary P value threshold to the binding data, and the heuristic methods ReMoDiscovery and GRAM used several arbitrary thresholds on both binding affinity and expression correlation coefficients to select regulatory targets. It is also worth noting that both COGRIM and each of these previous integrated approaches performed better than the method based on ChIP binding alone.

In addition to predicting sets of target genes, our COGRIM model also allows us to infer whether each TF acts as an activator or repressor, which we can compare with findings using previous methods. TFs that have significant positive effects *b*_*j *_on gene expression were classified as activators, whereas TFs that have significant negative *b*_*j*_s are defined as repressors. Significant effects were determined by examining whether the posterior interval for each *b*_*j *_overlapped with zero (details are given in Additional data file 1 [Supplementary methods]). In addition to agreement with the specific results of GRAM [4], this analysis identified seven more activators as well as one repressor RME1 (Additional data file 1 [Supplementary Table 6]). Five of the seven activators and the RME1 repressor discovered by our model were previously reported in the literature, which provides further evidence that our method is rather effective at distinguishing appropriate TF-regulon relationships when compared with GRAM. Moreover, the consistent correlations between TF expression and target gene expression support our assumption that the expression profiles of TF genes can act as a proxy for TF regulatory activity in many cases.

## Discussion

We have developed a statistical model to integrate different types of biologic information (gene expression data, ChIP binding data, and TF binding site data) in a flexible framework that allows genes to belong to multiple regulatory clusters. Our model was applied to available yeast data, resulting in more refined gene clusters than those derived from a single data source alone. We predict that roughly half of the TF target genes (B+/C-) predicted from ChIP binding data alone are not functional targets, and about 14% of genes (B-/C+) that were not identified based on ChIP binding data alone were predicted by our method to be functional target genes regulated by TFs. Our validation analyses indicate that these predicted novel targets are very likely to be functional TF target genes that are involved in relevant biologic pathways. Comparisons with several previous methods suggest that COGRIM is able to perform better on identifying appropriate functional regulatory targets. We also can use our model to integrate TF binding site data (from PWM scanning) and expression data when no ChIP binding data are available. For example, our application to the transcription factor SRF led to a reduced number of false-positive target gene predictions compared to the use of the PWM scan data alone. Finally, our study of C/EBP-β demonstrates that our model can integrate all three data types to identify functional gene targets in a principled way by estimating appropriate weights for the different data sources. Moreover, our studies on SRF and C/EBP-β demonstrate the effectiveness of our COGRIM model for applications in higher eukaryotic organisms.

The key aspect of our approach is that we include a probabilistic model for each data source, which addresses the inherent uncertainty within each data type. As a result, our model includes additional sources of data, contains fewer arbitrary thresholds, and does not require predefined gene clusters from a particular data source as compared with some previous integrated approaches [4,14]. Our probabilistic model also has advantages over the 'network component analysis' (NCA) approach [10-12], which assumes that the connectivity graph derived from ChIP binding data is known without error. Our results demonstrate that this assumption is often unrealistic, especially when one considers that ChIP experiments are typically limited to a single condition, but that TF binding can vary across different conditions. The tenuous assumption of a fixed connectivity graph allows the NCA approach more freedom to model TF activity directly. In comparison, our model focuses on direct estimation of the connectivity graph using multiple data sources (all with uncertainty), but it relies on a simplifying assumption regarding TF activity, namely that the activity of a TF depends on the expression of the gene encoding that TF. We acknowledge that this assumption will not hold in all cases, but our studies show that it is usually reasonable, especially given the limited amount of data on direct measurement of TF activities.

Our model and the NCA procedure can be regarded as complementary approaches to the same problem of network elucidation in the presence of both uncertain connectivity links and uncertain TF activity. However, our model has the additional advantage of utilizing both ChIP binding data and TF binding site data simultaneously when both are available, as well as estimating a weighting parameter that balances these two sources of data according to their relative uncertainty. The ability to weight these data sources optimally was demonstrated in the case of C/EBP-β. ChIP binding data and binding site predictions are intuitively related, and this is captured in Eqn 3. This methodology could also be used to balance the information from multiple ChIP experiments on the same TF when these data are available.

Our Bayesian hierarchical model is more than a simple extension of previous linear models in that it provides a principled mechanism for integrating ChIP binding and binding site data with expression data for prediction of target genes. Our model can be further extended in several interesting directions in the presence of additional data sources. If the available gene expression data come from time-course experiments, our model can be ameliorated with additional linear terms that would capture time delayed regulatory activities, such as modeling the expression of gene *i *at time *m *as a function of TF gene expression at not only time *m *but also at times *m *- 1, *m *- 2, and so on.

It should also be noted that although we have focused on TF proteins, our model would work equally well with regulatory factors that are not proteins but whose levels can be measured and whose binding sites can be identified (for example, microRNAs). This current work represents an initial step toward solving the problem of integrating available biological information in a principled fashion. Our belief is that this goal will best be accomplished by fitting large and flexible probability models that combine data from various experimental and compiled sources in a structured or multi-level framework. Despite the limitation due to the availability of expression profiles and the sensitivity of the various microarray platforms, we anticipate that our model will become even more valuable as the accuracy and coverage of expression and ChIP binding data improve.

## Materials and methods

The primary goal of our statistical model is to infer probable gene-TF interactions through the integration of available biologic data. Mathematically, we formulate our parameters of interest as unknown indicator variables *C*_*ij *_= 1 if gene *i *is regulated by TF *j *or 0 otherwise. Collectively, the matrix *C *of these indicator variables gives us our clusters of co-regulated genes, because all genes *i*, where *C*_*ij *_= 1, are estimated to be in a cluster together regulated by TF *j*. These indicator variables are the basis of a regulatory network, and can be visually represented as edges in a graph that connects TFs to the genes that they regulate. An important aspect of this formulation is that we are explicitly allowing genes to belong to multiple clusters controlled by different transcription factors (for instance, *C*_*ij *_= 1 and *C*_*ij*' _= 1 for *j *≠ *j'*).

In order to infer likely values for *C*, our model incorporates up to three general classes of biologic information: gene expression data, ChIP binding data, and sequence-level binding site data. We denote our expression data as *g*_*it*_, the log-expression of gene *i *(*I *= 1 ... *N*) in experiment *t *(*t *= 1 ... *T*). The set of *T *experiments can be from different tissues, different time course experiments, and different gene knockout experiments, or any combination thereof. Within these expression data, we give special focus on the expression of genes that encode known TF proteins. For our *J *known TFs, we denote *f*_*jt *_as the regulation activity currently estimated by log expression of TF gene *j *(*j *= 1 ... *J*) in experiment *t*. In addition to expression data, we have available ChIP experiments, which give information on the physical binding location of specific TFd. We use *b*_*ij *_to denote the probability that TF *j *physically binds in close proximity to gene *i*, from a ChIP binding experiment for TF *j*. Finally, we have available sequence-level information in the form of known or putative binding sites for specific TFs located in the upstream regions of target genes. We denote *m*_*ij *_as the probability that TF *j *has a binding site in the regulatory region of gene *i*. These binding sites could be experimentally verified, or predicted by scanning upstream sequences for similarity to an established PWM for a particular TF. We outline our model in the most general case, where all three of these data types are present, but we also discuss the ramifications on our procedure when only subsets of these data types are available.

Our model consists of multiple levels organized in a hierarchical fashion. The first level of our model incorporates our gene expression data by specifying the observed gene log expression *g*_*it *_as a linear function of TF gene log expression, *f*_*jt*_:

git=αi+∑j=1JβjCijfjt+εit   εit∼Normal(0,σ2)    (1)
 MathType@MTEF@5@5@+=feaafiart1ev1aaatCvAUfeBSjuyZL2yd9gzLbvyNv2Caerbhv2BYDwAHbqedmvETj2BSbqee0evGueE0jxyaibaiKI8=vI8tuQ8FMI8Gi=hEeeu0xXdbba9frFj0=OqFfea0dXdd9vqai=hGuQ8kuc9pgc9s8qqaq=dirpe0xb9q8qiLsFr0=vr0=vr0dc8meaabaqaciGacaGaaeqabaqadeqadaaakeaacaWGNbWaaSbaaSqaaiaadMgacaWG0baabeaakiabg2da9iabeg7aHnaaBaaaleaacaWGPbaabeaakiabgUcaRmaaqahabaGaeqOSdi2aaSbaaSqaaiaadQgaaeqaaaqaaiaadQgacqGH9aqpcaaIXaaabaGaamOsaaqdcqGHris5aOGaam4qamaaBaaaleaacaWGPbGaamOAaaqabaGccaWGMbWaaSbaaSqaaiaadQgacaWG0baabeaakiabgUcaRmrtHrhAqLwmaeHbnfgDOvwBHrxAJfgBqLgiXaaceiGae8xTdu2aaSbaaSqaaiaadMgacaWG0baabeaakiaaxMaacqWF1oqzdaWgaaWcbaGaamyAaiaadshaaeqaaOGaeSipIOJaamOtaiaad+gacaWGYbGaamyBaiaadggacaWGSbGaaiikaiaaicdacaGGSaGaeq4Wdm3aaWbaaSqabeaacaaIYaaaaOGaaiykaiaaxMaadaqadaqaaiaaigdaaiaawIcacaGLPaaaaaa@69D2@

We are using the expression *f*_*jt *_of the gene that encodes TF *j *as a proxy for the activity of TF *j*, in which case *b*_*j *_is the linear effect of TF *j *on target gene expression. Note that only TFs with probable connections to gene *i *(C_*ij *_= 1) are allowed to influence the expression of gene *i *in the above equation. This also means that *a*_*i *_can be interpreted as the baseline expression for gene *i *in the absence of regulation by known TFs (*C*_*ij *_= 0 for all TFs *j*). However, Eqn 1 (above) and similar linear models [6,10] are limited by not allowing for combinatorial relationships between TFs. Each TF *j *has a single effect (*b*_*j*_) on the expression of gene *i*, which does not take into account the biologic reality that expression is often the result of synergistic or antagonistic binding of multiple TFs. We acknowledge these combinatorial relationships by expanding our linear model to include interaction terms:

git=αi+∑j=1JβjCijfjt+∑j≠kγjkCijCikfjtfkt+εit  εit∼Normal(0,σ2)    (2)
 MathType@MTEF@5@5@+=feaafiart1ev1aaatCvAUfeBSjuyZL2yd9gzLbvyNv2Caerbhv2BYDwAHbqedmvETj2BSbqee0evGueE0jxyaibaiKI8=vI8tuQ8FMI8Gi=hEeeu0xXdbba9frFj0=OqFfea0dXdd9vqai=hGuQ8kuc9pgc9s8qqaq=dirpe0xb9q8qiLsFr0=vr0=vr0dc8meaabaqaciGacaGaaeqabaqadeqadaaakeaacaWGNbWaaSbaaSqaaiaadMgacaWG0baabeaakiabg2da9iabeg7aHnaaBaaaleaacaWGPbaabeaakiabgUcaRmaaqahabaGaeqOSdi2aaSbaaSqaaiaadQgaaeqaaaqaaiaadQgacqGH9aqpcaaIXaaabaGaamOsaaqdcqGHris5aOGaam4qamaaBaaaleaacaWGPbGaamOAaaqabaGccaWGMbWaaSbaaSqaaiaadQgacaWG0baabeaakiabgUcaRmaaqafabaGaeq4SdC2aaSbaaSqaaiaadQgacaWGRbaabeaakiaadoeadaWgaaWcbaGaamyAaiaadQgaaeqaaOGaam4qamaaBaaaleaacaWGPbGaam4AaaqabaGccaWGMbWaaSbaaSqaaiaadQgacaWG0baabeaakiaadAgadaWgaaWcbaGaam4AaiaadshaaeqaaOGaey4kaScaleaacaWGQbGaeyiyIKRaam4Aaaqab0GaeyyeIuoat0uy0HwzTfgDPnwyXaqeg0uy0HwzTfgDPnwyXaaceiGccqWF1oqzdaWgaaWcbaGaamyAaiaadshaaeqaaOGaaeiiaiaabccacqWF1oqzdaWgaaWcbaGaamyAaiaadshaaeqaaOGaeSipIOJaamOtaiaad+gacaWGYbGaamyBaiaadggacaWGSbGaaiikaiaaicdacaGGSaGaeq4Wdm3aaWbaaSqabeaacaaIYaaaaOGaaiykaiaaxMaadaqadaqaaiaaikdaaiaawIcacaGLPaaaaaa@8188@

Where we now have additional coefficients *g*_*jk*_, which can be interpreted as the synergistic (or antagonistic) effect of both TFs *j *and *k *binding together to the same upstream region (in addition to the effects of TF *j *or *k *binding in isolation). Considering the large number of factors involved in these linear equations, we should be cautious about over-interpretation of individual *b*_*j *_or *g*_*jk *_coefficients, but these parameters can still provide information about the partial effects of particular TFs on gene expression. It should also be noted that the gene expression data we use is on the log scale, and although this same model could be used for measurements of absolute expression levels (when available), the interpretation of the linear and interaction terms would be quite different in that situation. Our model could also be expanded to allow higher order interaction terms, although at increased computational cost.

As mentioned above, we may have two additional classes of data for a particular gene-TF interaction *C*_*ij*_. We may have *b*_*ij*_, the probability that TF *j *physically binds in proximity to gene *i *in a ChIP binding experiment, and *m*_*ij*_, the probability of a binding site for TF *j *in the upstream region of gene *i*. The second level of our model incorporates both *b*_*ij *_and *m*_*ij *_into a prior distribution for our unknown indicator variable *C*_*ij*_. For a given value of the weight variable *w*_*j*_, the distribution of our clustering indicators *C*_*ij *_given *b*_*ij *_and *m*_*ij *_can be factored into a product of two prior distributions, one based on *b*_*ij *_and one based on *m*_*ij*_.

p(Cij|mij,bij,wj)∝[bijCij(1−bij)1−Cij]wj⋅[mijCij(1−mij)1−Cij]1−wj     (3)
 MathType@MTEF@5@5@+=feaafiart1ev1aaatCvAUfeBSjuyZL2yd9gzLbvyNv2Caerbhv2BYDwAHbqedmvETj2BSbqee0evGueE0jxyaibaiKI8=vI8tuQ8FMI8Gi=hEeeu0xXdbba9frFj0=OqFfea0dXdd9vqai=hGuQ8kuc9pgc9s8qqaq=dirpe0xb9q8qiLsFr0=vr0=vr0dc8meaabaqaciGacaGaaeqabaqadeqadaaakeaacaWGWbGaaiikaiaadoeadaWgaaWcbaGaamyAaiaadQgaaeqaaOGaaiiFaiaad2gadaWgaaWcbaGaamyAaiaadQgaaeqaaOGaaiilaiaadkgadaWgaaWcbaGaamyAaiaadQgaaeqaaOGaaiilaiaadEhadaWgaaWcbaGaamOAaaqabaGccaGGPaGaeyyhIu7aamWaaeaacaWGIbWaa0baaSqaaiaadMgacaWGQbaabaGaam4qamaaBaaameaacaWGPbGaamOAaaqabaaaaOGaaiikaiaaigdacqGHsislcaWGIbWaaSbaaSqaaiaadMgacaWGQbaabeaakiaacMcadaahaaWcbeqaaiaaigdacqGHsislcaWGdbWaaSbaaWqaaiaadMgacaWGQbaabeaaaaaakiaawUfacaGLDbaadaahaaWcbeqaaiaadEhadaWgaaadbaGaamOAaaqabaaaaOGaeyyXIC9aamWaaeaacaWGTbWaa0baaSqaaiaadMgacaWGQbaabaGaam4qamaaBaaameaacaWGPbGaamOAaaqabaaaaOGaaiikaiaaigdacqGHsislcaWGTbWaaSbaaSqaaiaadMgacaWGQbaabeaakiaacMcadaahaaWcbeqaaiaaigdacqGHsislcaWGdbWaaSbaaWqaaiaadMgacaWGQbaabeaaaaaakiaawUfacaGLDbaadaahaaWcbeqaaiaaigdacqGHsislcaWG3bWaaSbaaWqaaiaadQgaaeqaaaaakiaaxMaacaWLjaWaaeWaaeaacaaIZaaacaGLOaGaayzkaaaaaa@755C@

The variable *w*_*j *_is the relative weight of the prior ChIP-binding information *b*_*ij *_versus the TF binding site information *m*_*ij*_. The weights *w *= (*w*_1 _... *w*_*J*_) are TF specific but not gene specific, and are designed to reflect potential global differences in quality between the binding data and PWM scanning data for TF *j*. However, because this relative quality is not necessarily known *a priori*, we will treat each weight *w*_*j *_as an unknown variable. Clearly, if only ChIP binding data for TF *j *are available then *w*_*j *_= 1 and Eqn 3 reduces to a function of *b*_*ij *_only, whereas if only TF binding site data for TF *j *is available then *w*_*j *_= 0 and Eqn 3 reduces to a function of *m*_*ij *_only. Our weighted prior methodology could also be used to balance the information from two different ChIP binding experiments, in which case the variable weight would measure the relative quality between the two ChIP datasets. It would also be easy to extend our model to accommodate more than two sources of data into our prior distribution, which would involve the use of multiple weight variables between the different data sources. Often, the available ChIP binding or TF binding site data are not available directly as probabilities, but rather as *P *values or scores from a previous statistical analysis. We convert these *P *values or scores to probabilities with an EM (expectation maximization) algorithm [30] based on a mixture model, which is described in detail in the Additional data file 1 (Supplementary Materials).

The Bayesian paradigm gives us a principled framework for connecting these model levels into a single posterior distribution for all unknown parameters:

*p*(*C*,*w*,Θ|*g*,*f*,*m*,*b*) ∝ *p*(*g*|*f*,*C*,Θ)·*p*(*C*|*m*,*b*,*w*)·*p*(Θ,*w*)

where Θ denotes the collection of linear model parameters (Θ = α,β,γ,σ^2 ^...). The term *p*(*g*|*f*,*C*,Θ) represents our first model level with expression data *g *= (*g*_*it*_) and *f *= (*f*_*jt*_), and *p*(*C*|*m*,*b*,*w*) represents our second model level with ChIP binding data *b *= (*b*_*ij*_) and TF binding site data *m *= (*m*_*ij*_). All that remains is the specification *p*(Θ,*w*), the prior distributions for our TF specific prior weights *w *= (*w*_1 _... *w*_*J*_) and our linear model parameters Θ (summarized in Table 5).

In the absence of additional prior information about these parameters, we can make these prior distributions 'non-informative' (non-influential relative to the data) by setting our prior variance parameters τα2
 MathType@MTEF@5@5@+=feaafiart1ev1aaatCvAUfeBSjuyZL2yd9gzLbvyNv2Caerbhv2BYDwAHbqedmvETj2BSbqee0evGueE0jxyaibaiKI8=vI8tuQ8FMI8Gi=hEeeu0xXdbba9frFj0=OqFfea0dXdd9vqai=hGuQ8kuc9pgc9s8qqaq=dirpe0xb9q8qiLsFr0=vr0=vr0dc8meaabaqaciGacaGaaeqabaqadeqadaaakeaacqaHepaDdaqhaaWcbaGaeqySdegabaGaaGOmaaaaaaa@377A@, τβ2
 MathType@MTEF@5@5@+=feaafiart1ev1aaatCvAUfeBSjuyZL2yd9gzLbvyNv2Caerbhv2BYDwAHbqedmvETj2BSbqee0evGueE0jxyaibaiKI8=vI8tuQ8FMI8Gi=hEeeu0xXdbba9frFj0=OqFfea0dXdd9vqai=hGuQ8kuc9pgc9s8qqaq=dirpe0xb9q8qiLsFr0=vr0=vr0dc8meaabaqaciGacaGaaeqabaqadeqadaaakeaacqaHepaDdaqhaaWcbaGaeqOSdigabaGaaGOmaaaaaaa@377C@ and τγ2
 MathType@MTEF@5@5@+=feaafiart1ev1aaatCvAUfeBSjuyZL2yd9gzLbvyNv2Caerbhv2BYDwAHbqedmvETj2BSbqee0evGueE0jxyaibaiKI8=vI8tuQ8FMI8Gi=hEeeu0xXdbba9frFj0=OqFfea0dXdd9vqai=hGuQ8kuc9pgc9s8qqaq=dirpe0xb9q8qiLsFr0=vr0=vr0dc8meaabaqaciGacaGaaeqabaqadeqadaaakeaacqaHepaDdaqhaaWcbaGaeq4SdCgabaGaaGOmaaaaaaa@3782@ to be very large (in this study, 10,000) and setting the degrees of freedom for σ^2 ^to be small (in this study, 2). This complicated model is implemented using Gibbs sampling [31], which is an iterative Markov Chain Monte Carlo algorithm that samples new values for each set of unknown parameters conditional on the current values of all other parameters. The COGRIM R package and supplementary materials are available for download from our COGRIM website [32].

## Additional data files

The following additional data are available with the online version of this paper. Additional data file 1 includes our detailed model procedures, Gibbs sampling algorithm, data processing procedures, predicted gene targets, and annotation evidence. Additional data file 2 is the COGRIM R program.

## Supplementary Material

Additional data file 1 Detailed model procedures, Gibbs sampling algorithm, data processing procedures, predicted gene targets, and annotation evidence. Click here for file

Additional data file 2 COGRIM R program. Click here for file

## Figures and Tables

**Table 1:** Gene classification from ChIP binding data and expression data

TF	B+		B-	
	
	B+/C-	B+/C+	B-/C+	B-/C-
ACE2	46	22	9	5964
SWI4	25	99	36	5881
SWI5	54	40	22	5925
SWI6	54	39	48	5900
MBP1	48	56	29	5908
STB1	6	17	15	6003
SKN7	49	46	26	5920
FKH1	36	26	45	5934
FKH2	59	46	48	5888
NDD1	19	74	10	5938
MCM1	44	42	31	5924
ABF1	99	175	128	5639
BAS1	34	8	17	5982
CAD1	28	10	10	5993
CBF1	24	19	28	5970
GAL4	12	28	3	5998
GCN4	26	53	11	5951
GCR1	6	6	10	6019
GCR2	23	8	15	5995
HAP2	4	14	23	6000
HAP3	11	11	16	6003
HAP4	33	34	17	5957
HSF1	34	18	55	5934
INO2	5	6	14	6016
LEU3	15	6	22	5998
MET31	21	6	31	5983
MSN4	24	6	13	5998
PDR1	22	44	19	5956
PHO4	36	23	19	5963
PUT3	3	6	0	6032
RAP1	113	87	64	5777
RCS1	16	15	19	5991
REB1	67	72	59	5843
RLM1	23	14	12	5992
RME1	13	3	15	6010
ROX1	20	9	20	5992
SMP1	24	39	16	5962
STE12	33	17	28	5963
YAP1	27	17	21	5976

A total of 6041 ORFs are considered, based on availability of expression data and binding data, and 1542 target genes are selected in C+ (B+/C+ and B-/C+) by applying a posterior probability cutoff of 0.5 on each *C*_*ij *_(see COGRIM website [32] for the lists of gene ORFs for each TF). ORF, open reading frame; TF, transcription factor.

**Table 2:** Regulatory response to transcription factor deletion

	Genome wide		B+/C+			B+/C-			B-/C+		
	
	Mean	SD	Mean	SD	*P *value	Mean	SD	*P *value	Mean	SD	*P *value
Yap1	0.003	0.092	-0.174	0.23	7.24 × e^-03^	0.043	0.13	0.158	-0.104	0.16	6.31 × e^-03^
Swi5	0.004	0.06	-0.1	0.166	3.71 × e^-04^	0.006	0.042	0.668	-0.019	0.03	1.01 × e^-03^
Swi4	0.015	0.17	-0.124	0.24	1.23 × e^-07^	0.058	0.178	0.242	-0.067	0.156	3.29 × e^-03^
Gcn4	-0.007	0.07	-0.26	0.22	2.57 × e^-11^	-0.002	0.032	0.421	-0.158	0.137	4.40 × e^-03^

By conducting standard *t*-tests, the significance of the change in expression between knockout and wild-type was examined within each gene set (B+/C+, B-/C+, B+/C-) for four transcription factors for which expression, ChIP-ChIP, and deletion data are available. ChIP, chromatin immunoprecipitation; SD, standard deviation.

**Table 3:** Comparison with previous approaches based on MIPS category enrichment and expression correlation coefficients

Method	Average percentage genes in enriched MIPS categories	Average expression correlation coefficient
COGRIM (B+/C+)	0.450	0.341
COGRIM (B-/C+)	0.349	0.380
Model I (B+/C+)	0.401	0.340
Model I (B-/C+)	0.340	0.372
MA-Networker	0.338	0.171
GRAM	0.352	0.337
ReMoDiscovery	0.347	0.291
ChIP binding data alone (B+)	0.217	0.165

'Average percentage genes in enriched MIPS categories' is the percentage of genes with enriched MIPS categories, averaged over all the 39 yeast TFs. Model I, COGRIM without interaction terms; TF, transcription factor.

**Table 4:** Comparison with previous approaches based on gene expression response to TF deletion

Method	YAP1	SWI5	SWI4	GCN4
COGRIM (B+/C+)	7.24 × e^-03^	3.71 × e^-04^	1.23 × e^-07^	2.57 × e^-11^
COGRIM (B-/C+)	6.31 × e^-03^	1.01 × e^-03^	3.29 × e^-03^	4.40 × e^-03^
Model I (B+/C+)	0.018	4.00 × e^-04^	7.31 × e^-04^	4.80 × e^-09^
Model I (B-/C+)	0.012	8.14 × e^-03^	1.07 × e^-03^	7.95 × e^-03^
MA-Networker	0.021	1.81 × e^-04^	2.34 × e^-06^	2.17 × e^-10^
GRAM	0.259	0.281	1.14 × e^-05^	1.54 × e^-04^
ReMoDiscovery	0.102	7.73 × e^-03^	0.364	3.23 × e^-10^
ChIP binding data alone (B+)	0.194	0.036	9.80 × e^-04^	1.96 × e^-04^

Standard *t*-tests were conducted to indicate the significance of the change in expression between knockout and wild-type. Model I, COGRIM without interaction terms; TF, transcription factor.

**Table 5:** Linear model parameters

Parameter	Details
Baseline gene *i *expression	α_i _~ *Normal*(0,τα2 MathType@MTEF@5@5@+=feaafiart1ev1aaatCvAUfeBSjuyZL2yd9gzLbvyNv2Caerbhv2BYDwAHbqedmvETj2BSbqee0evGueE0jxyaibaiKI8=vI8tuQ8FMI8Gi=hEeeu0xXdbba9frFj0=OqFfea0dXdd9vqai=hGuQ8kuc9pgc9s8qqaq=dirpe0xb9q8qiLsFr0=vr0=vr0dc8meaabaqaciGacaGaaeqabaqadeqadaaakeaacqaHepaDdaqhaaWcbaGaeqySdegabaGaaGOmaaaaaaa@377A@)
TF linear effects	β = (β_1_, ..., β_J_) ~ *MultivariateNormal*(0,*I*·τβ2 MathType@MTEF@5@5@+=feaafiart1ev1aaatCvAUfeBSjuyZL2yd9gzLbvyNv2Caerbhv2BYDwAHbqedmvETj2BSbqee0evGueE0jxyaibaiKI8=vI8tuQ8FMI8Gi=hEeeu0xXdbba9frFj0=OqFfea0dXdd9vqai=hGuQ8kuc9pgc9s8qqaq=dirpe0xb9q8qiLsFr0=vr0=vr0dc8meaabaqaciGacaGaaeqabaqadeqadaaakeaacqaHepaDdaqhaaWcbaGaeqOSdigabaGaaGOmaaaaaaa@377C@)
TF interaction effects	γ = (γ_12_, ..., γ_JK_) ~ *MultivariateNormal*(0,*I*·τγ2 MathType@MTEF@5@5@+=feaafiart1ev1aaatCvAUfeBSjuyZL2yd9gzLbvyNv2Caerbhv2BYDwAHbqedmvETj2BSbqee0evGueE0jxyaibaiKI8=vI8tuQ8FMI8Gi=hEeeu0xXdbba9frFj0=OqFfea0dXdd9vqai=hGuQ8kuc9pgc9s8qqaq=dirpe0xb9q8qiLsFr0=vr0=vr0dc8meaabaqaciGacaGaaeqabaqadeqadaaakeaacqaHepaDdaqhaaWcbaGaeq4SdCgabaGaaGOmaaaaaaa@3782@)
Residual gene expression variance	σ^2 ^~ *Inv*-χ22 MathType@MTEF@5@5@+=feaafiart1ev1aaatCvAUfeBSjuyZL2yd9gzLbvyNv2Caerbhv2BYDwAHbqedmvETj2BSbqee0evGueE0jxyaibaiKI8=vI8tuQ8FMI8Gi=hEeeu0xXdbba9frFj0=OqFfea0dXdd9vqai=hGuQ8kuc9pgc9s8qqaq=dirpe0xb9q8qiLsFr0=vr0=vr0dc8meaabaqaciGacaGaaeqabaqadeqadaaakeaacqaHhpWydaqhaaWcbaGaaGOmaaqaaiaaikdaaaaaaa@3689@ (inverse Chi-square)
Prior distribution weights	*w*_*j *_~ *Uniform*(0,1)

TF, transcription factor.
