# Supplementary material for: Clustering of genes into regulons using integrated modeling-COGRIM
Source: Genome Biol. 2007 Jan 4;8(1):R4. doi: 10.1186/gb-2007-8-1-r4 (PMC1839128; doi:10.1186/gb-2007-8-1-r4)

Supplementary Methods

1 Parameter Estimation and Inference

The posterior distribution of our unknown parameters is proportional to the product of our model likelihood and our assumed prior distributions,

$$
\begin{aligned}
p(\mathbf{C}, \mathbf{w}, \boldsymbol{\Theta}|\mathbf{g}, \mathbf{f}, \mathbf{m}, \mathbf{b}) &\propto p(\mathbf{g}|\mathbf{f}, \mathbf{C}, \boldsymbol{\Theta}) \cdot p(\mathbf{C}|\mathbf{m}, \mathbf{b}, \mathbf{w}) \cdot p(\mathbf{w}) \cdot p(\boldsymbol{\alpha}) \cdot p(\boldsymbol{\beta}) \cdot p(\sigma^2) \\
&\propto \prod_{i=1}^{N}\prod_{t=1}^{T}(2\pi\sigma^2)^{-1/2} \exp\left[\frac{-1}{2\sigma^2}\left(g_{it} - \alpha_i - \sum_{j=1}^{J}\beta_j X_{ijt}\right)^2\right] \\
&\cdot \prod_{i=1}^{N}\prod_{j=1}^{J}\left[b_{ij}^{C_{ij}}(1-b_{ij})^{1-C_{ij}}\right]^{w_j} \cdot \left[m_{ij}^{C_{ij}}(1-m_{ij})^{1-C_{ij}}\right]^{1-w_j} \\
&\cdot \prod_{i=1}^{N}(\tau_\alpha^2)^{-1/2} \exp\left[\frac{-1}{2\tau_\alpha^2}\alpha_i^2\right] \cdot \prod_{j=1}^{J}(\tau_\beta^2)^{-1/2} \exp\left[\frac{-1}{2\tau_\beta^2}\beta_j^2\right] \cdot (\sigma^2)^{-2} \exp\left[\frac{-1}{2\sigma^2}\right]
\end{aligned}
$$

where $X_{ijt} = C_{ij} f_{jt}$. We estimate all unknown parameters by Gibbs sampling, ie. by iteratively estimating one set of parameters given all other parameters. Specifically, we iterate between

1. Estimating $\boldsymbol{\Theta}$ given $\mathbf{C}, \mathbf{w}$ and data $\mathbf{g}, \mathbf{f}, \mathbf{b}, \mathbf{m}$
2. Estimating $\mathbf{C}$ given $\mathbf{w}, \boldsymbol{\Theta}$ and data $\mathbf{g}, \mathbf{f}, \mathbf{b}, \mathbf{m}$
3. Estimating $\mathbf{w}$ given $\mathbf{C}, \boldsymbol{\Theta}$ and data $\mathbf{g}, \mathbf{f}, \mathbf{b}, \mathbf{m}$

The details of these steps are given in the following sections. The result of this Gibbs sampling algorithm is a set of values for each parameter from the posterior distribution given above. In this study, we use these posterior parameter values for inference in two different ways. First, the parameter values for each C_{ij} are used to define whether gene i is actually regulated by TF j according to our model: if the majority of parameter values for C_{ij} are $C_{ij} = 1$ (as opposed to $C_{ij} = 0$), then we claim that gene i is regulated by TF j. This gene is then placed in the **C+** group for TF j. Secondly, we use the parameter values for our linear effects $\boldsymbol{\beta}$ and interaction effects $\boldsymbol{\gamma}$ to form 95% posterior intervals for each parameter β_j and γ_{jk} by excluding the most extreme 2.5% of the parameter draws in either direction. If the posterior interval for a particular β_j does not contain zero, we conclude that there is significant linear effect for TF j. Similarly, if the posterior interval for a particular γ_{jk} does not contain zero, we concluded that there is a significant interaction effect between the TFs j and k.

2 Algorithm Step 1: Estimating linear model parameters $\boldsymbol{\Theta}$

The regulation matrix $\mathbf{C}$ is assumed known during this step, so we do not need to use our prior data $\mathbf{b}, \mathbf{m}$ or the current values of $\mathbf{w}$. We use $\mathbf{C}$ to construct the variables $\mathbf{X}$ where$X_{ijt} = C_{ij} f_{jt}$. The linear model parameters $\boldsymbol{\Theta}$ are then separately estimated by the following iterative strategy.

a. **Estimate α_i given $\boldsymbol{\beta}, \sigma^2$ and data (g's and X's):**
Since each intercept α_i is independent from the other α's, they can be separately sampled,

$$
\begin{aligned}
p(\alpha_i|\boldsymbol{\beta}, \sigma^2, \mathbf{g}, \mathbf{X}) &\propto \exp\left[\frac{-1}{2\sigma^2}\sum_{t=1}^{T}\left(g_{it} - \alpha_i - \sum_{j=1}^{J}\beta_j X_{ijt}\right)^2\right] \cdot \exp\left[\frac{-1}{2\tau_\alpha^2}\alpha_i^2\right] \\
&\propto \exp\left[\frac{-1}{2\nu_\alpha}\left(\alpha_i - \frac{\nu_\alpha}{\sigma^2} \cdot \sum_{t=1}^{T} Y_t\right)^2\right]
\end{aligned}
$$

where $Y_t = g_{it} - \sum_{j=1}^{J} \beta_j X_{ijt}$ and $\nu_\alpha = (T/\sigma^2 + 1/\tau_\alpha^2)^{-1}$. This distribution implies that

$$\alpha_i \sim \text{Normal}\left(\frac{\nu_\alpha}{\sigma^2} \cdot \sum_{t=1}^{T} Y_t \;,\; \nu_\alpha\right)$$

We can make our prior distribution for each α_i to be non-informative by making τ_α very large (in this study, 10000) relative to the contribution of the likelihood to the variance (σ^2/T).

b. **Estimate β_j given $\boldsymbol{\alpha}, \sigma^2$ and data (g's and X's).**
Note that in the step below, we have combined our notation for interaction coefficients γ_{jk} and linear coefficients β_j into a single vector of parameters $\boldsymbol{\beta}$. These coefficients β_j are not independent from each other, and so must be iteratively sampled themselves:

$$\begin{aligned} p(\beta_j|\boldsymbol{\alpha}, \sigma^2, \mathbf{g}, \mathbf{X}) &\propto \exp\left[\frac{-1}{2\sigma^2}\sum_{i=1}^{N}\sum_{t=1}^{T}\left(g_{it} - \alpha_i - \sum_{j'=1}^{J}\beta_{j'}X_{ij't}\right)^2\right] \cdot \exp\left[\frac{-1}{2\tau_\beta^2}\beta_j^2\right] \\ &\propto \exp\left[\frac{-1}{2\sigma^2}\sum_{i=1}^{N}\sum_{t=1}^{T}(V_{it} - \beta_j X_{ijt})^2\right] \cdot \exp\left[\frac{-1}{2\tau_\beta^2}\beta_j^2\right] \end{aligned}$$

where $V_{it} = g_{it} - \alpha_i - \sum_{j' \neq j} \beta_{j'} X_{ij't}$, which reduces further to

$$p(\beta_j|\boldsymbol{\alpha}, \sigma^2, \mathbf{g}, \mathbf{X}) \propto \exp\left[\frac{-1}{2\nu_\beta}\left(\beta_j - \frac{\nu_\beta}{\sigma^2} \cdot T_{VX}\right)^2\right]$$

where $\nu_\beta = (T_{XX}/\sigma^2 + 1/\tau_\beta^2)^{-1}$, $T_{XX} = \sum_{i=1}^{N}\sum_{t=1}^{T} X_{ijt}^2$ and $T_{VX} = \sum_{i=1}^{N}\sum_{t=1}^{T} V_{it}X_{ijt}$.
This distribution implies that

$$\beta_j \sim \text{Normal}\left(\frac{\nu_\beta}{\sigma^2} \cdot T_{VX} \;,\; \nu_\beta\right)$$

Note that the distribution of β_j depends on the values of the other $\beta_{j'}$'s, so we must sample the β_j's one at a time given the current values of the other $\beta_{j'}$'s. We can make our prior distribution for each β_j to be non-informative by making τ_β very large (in this study, 10000) relative to the contribution of the likelihood to the variance (σ^2/T_{XX}).

c. **Estimate σ^2 given $\boldsymbol{\beta}, \boldsymbol{\alpha}$ and data (g's and X's).**
For the residual variance σ^2, we have the following conditional distribution

$$\begin{aligned} p(\sigma^2|\boldsymbol{\alpha}, \boldsymbol{\beta}, \mathbf{g}, \mathbf{X}) &\propto (\sigma^2)^{-(\frac{TN}{2}+2)} \cdot \exp\left[\frac{-1}{2\sigma^2}\sum_{i=1}^{N}\sum_{t=1}^{T}\left(g_{it} - \alpha_i - \sum_{j=1}^{J}\beta_j X_{ijt}\right)^2\right] \cdot \exp\left(\frac{-1}{2\sigma^2}\right) \\ &\propto (\sigma^2)^{-(\frac{TN+2}{2}+1)} \cdot \exp\left[\frac{-1}{2\sigma^2}(V_\sigma + 1)\right] \end{aligned}$$

where $V_\sigma = \sum_{i=1}^{N}\sum_{t=1}^{T}(g_{it} - \alpha_i - \sum_{j=1}^{J}\beta_j X_{ijt})^2$. The above calculations use a χ_ν^2 prior distribution for σ with hyper-parameter $\nu = 2$. We see that the influence of this prior is very small on the posterior distribution for σ^2, which is a scaled-inverse χ^2 distribution with degrees of freedom parameter $TN + 2$ and scale parameter $s^2 = (V_\alpha + 1)/(TN + 2)$.

3 Algorithm Step 2: Estimating regulation matrix $\mathbf{C}$

We are assuming that both our linear model parameters $\mathbf{\Theta}$ and our weights $\mathbf{w}$ are known for this step of the algorithm. When estimating a new value for each C_{ij}, we also can condition on $\mathbf{C}'$, which is all the other $C_{i'j'}$ values in $\mathbf{C}$ ($i' \neq i$ and $j' \neq j$). This gives us the following conditional distribution for C_{ij}:

$$p(C_{ij}|\mathbf{\Theta}, \mathbf{w}, \mathbf{C}', \mathbf{g}, \mathbf{f}, \mathbf{b}, \mathbf{m}) \propto \exp\left[\frac{-1}{2\sigma^2}\sum_{t=1}^{T}\left(g_{it} - \alpha_i - \sum_{j=1}^{J}\beta_j C_{ij} f_{jt}\right)^2\right] \cdot \left[b_{ij}^{C_{ij}}(1-b_{ij})^{1-C_{ij}}\right]^{w_j} \cdot \left[m_{ij}^{C_{ij}}(1-m_{ij})^{1-C_{ij}}\right]^{1-w_j} \quad (1)$$

Let Z_1 be the value of equation (1) when $C_{ij} = 1$ and Z_0 be the value of equation (1) when $C_{ij} = 0$. We sample a new value of C_{ij} as follows:

$$C_{ij} = \begin{cases} 1 & \text{with probability } Z_1^\star \\ 0 & \text{with probability } Z_0^\star \end{cases}$$

where $Z_i^\star = Z_i/(Z_1 + Z_0)$. Moreover, to understand the contribution from expression data, we can design COGRIM to update the indicator C_{ij} without the ChIP binding and motif priors (b_{ij} and m_{ij}).

4 Algorithm Step 3: Estimating prior weights $\mathbf{w}$

We are assuming that the regulation matrix $\mathbf{C}$ is known for this step of the algorithm, so we do not need to use any of the expression data , $\mathbf{g}$ or linear model parameters $\mathbf{\Theta}$ at this point. For each TF j, we need to estimate a new weight w_j based on the following distribution:

$$p(w_j|\mathbf{C}, b_{ij}, m_{ij}) \propto \left[\prod_{i=1}^{n}(b_{ij})^{C_{ij}}(1-b_{ij})^{1-C_{ij}}\right]^{w_j} \cdot \left[\prod_{i=1}^{n}(m_{ij})^{C_{ij}}(1-m_{ij})^{1-C_{ij}}\right]^{1-w_j} \quad (2)$$

Note that there is also a normalizing constant present in (2) that is also a function of w_j, which comes from the integration of $p(w_j, \mathbf{C}_j|\mathbf{b}, \mathbf{m})$ over all possible values of $\mathbf{C}_j$. There is no built-in function for sampling a value from equation (2), but instead we can use *grid sampling*, as follows:

1. Set up a grid of n possible w_j values: $\mathbf{z} = (z_1 = 0.01, z_2 = 0.02, \ldots, z_n = 0.99)$
2. Evaluate equation (2) for each of these grid values. In other words, calculate

$$p_i = p(z_i|\mathbf{C}, b_{ij}, m_{ij})$$

3. Normalize the probabilities for each grid value: $p_i^\star = p_i / \sum_i p_i$
4. Set $w_j = z_i$ with probability $p_i^\star$

5 Evaluating Convergence

Multiple chains of our Gibbs sampling algorithm were run from different starting points. We monitored the convergence based on the within and between variance ratio $\hat{R}$ [34], which converges towards 1 as the multiple chains converge towards each other. The maximum $\hat{R}$ values (across different parameter sets) after 5000 iterations are shown in the following table for the three applications to Yeast, C/EBP-β and SRF. We concluded, based on these $\hat{R}$ values, that our Gibbs sampler chains had converged after 5000 iterations in each application. We also examined the autocorrelation function of the sampled draws. We observed a rapid decline in the autocorrelation for increasing lags which indicates that the sampler does not have a substantively high autocorrelation(data not shown).

	Yeast	C/EBP-β	SRF
$\hat{R}(\sigma^2)$	1.001	1.0002	1.0004
$\max_i \hat{R}(\alpha_i)$	1.026	1.011	1.008
$\max_j \hat{R}(\beta_j)$	1.014	1.005	1.002
$\max_j \hat{R}(w_j)$		1.007	

6 Permutation evaluation of the sensitivity

To understand the classification sensitivity on priors and posterior thresholds, we can employ a permutation scheme to make sure that our threshold of 0.5 is reasonable. Prior data sources b_{ij} obtained from ChIP binding data can be randomly permuted and then implemented in COGRIM to be compared with our results obtained using our actual non-permuted priors. This comparison can help us understand the sensitivity of our results to both prior information and posterior thresholds. As an example, we conducted this permutation analysis on a dataset for the HAP4 transcription factor in Yeast. The comparisons showed that (data not shown) (1) inference based on permuted prior information is much more sensitive to different posterior cutoffs than our actual results and (2) both the data in our prior distributions and the likelihood of our expression data are contributing substantially to our inferred target genes.

7 Converting Binding p-values into Binding Probabilities

The results produced by a typical ChIP binding experiment for TF j is a set of measures Y_i for the enrichment of each gene i for that TF j (often these measures are averages calculated over several repetitions of the experiment). These measures are typically standardized, $X_i = (Y_i - \overline{Y})/s_Y$, to have a common mean and standard deviation. For each X_i, a significance test is performed against a null hypothesis of no enrichment, giving a p-value p_i for each gene that is calculated using a standard normal or a t distribution. These p-values can not be directly interpreted as the probability $b_i = \mathrm{P}(\mathrm{TF}\ j \text{ binds gene } i)$, so we use the following procedure to convert p_i into binding probabilities b_i (Since b_{ij} is calculated for each TF j independently, we here just use i notation instead of ij for simplicity).

We first convert the p-values p_i back to their corresponding standardized enrichment measures X_i using the inverse-CDF for the standard normal distribution. The distribution of these enrichment measures X_i should be a mixture of two different groups: a large group of unenriched genes that should be centered at $X = 0$ and a smaller group of genes that are truly enriched, with center $\mu > 0$. This mixture model is visually represented for the transcription factor ABF1 in the following Figure.

Statistically, we can model each gene with a latent variable I_i that indicates whether that gene is in the enriched group ($I_i = 1$) or unenriched group ($I_i = 0$). Our desired binding probabilities for each gene are then simply $b_i = \mathrm{P}(I_i = 1)$. An EM algorithm [30] was written to simultaneously estimate the unknown parameters of our mixture model (mixing proportion and mean/variance of enriched component) as well as the $b_i = \mathrm{P}(I_i = 1)$ for each gene. This algorithm alternates between

1. estimating the unknown parameters of our mixture model given current estimates of each b_i:

$$\hat{\mu} = \frac{\sum_i I_i \cdot X_i}{\sum_i I_i} \qquad \hat{\tau}^2 = \frac{\sum_i I_i \cdot (X_i - \mu)^2}{\sum_i I_i} \qquad \hat{\lambda} = \frac{\sum_i I_i}{n}$$

2. estimating the unknown b_i's given current estimates $\hat{\mu}$ and $\hat{\lambda}$ using Bayes rule:

$$\begin{aligned} b_i = P(I_i = 1|X_i) &= \frac{P(X_i|I_i = 1)P(I_i = 1)}{P(X_i|I_i = 1)P(I_i = 1) + P(X_i|I_i = 0)P(I_i = 0)} \\ &= \frac{\hat{\tau}^{-1}\exp(\frac{1}{2\hat{\tau}^2}(X_i - \hat{\mu})^2) \cdot \hat{\lambda}}{\hat{\tau}^{-1}\exp(\frac{1}{2\hat{\tau}^2}(X_i - \hat{\mu})^2) \cdot \hat{\lambda} + \exp(\frac{1}{2}X_i^2) \cdot (1 - \hat{\lambda})} \end{aligned}$$

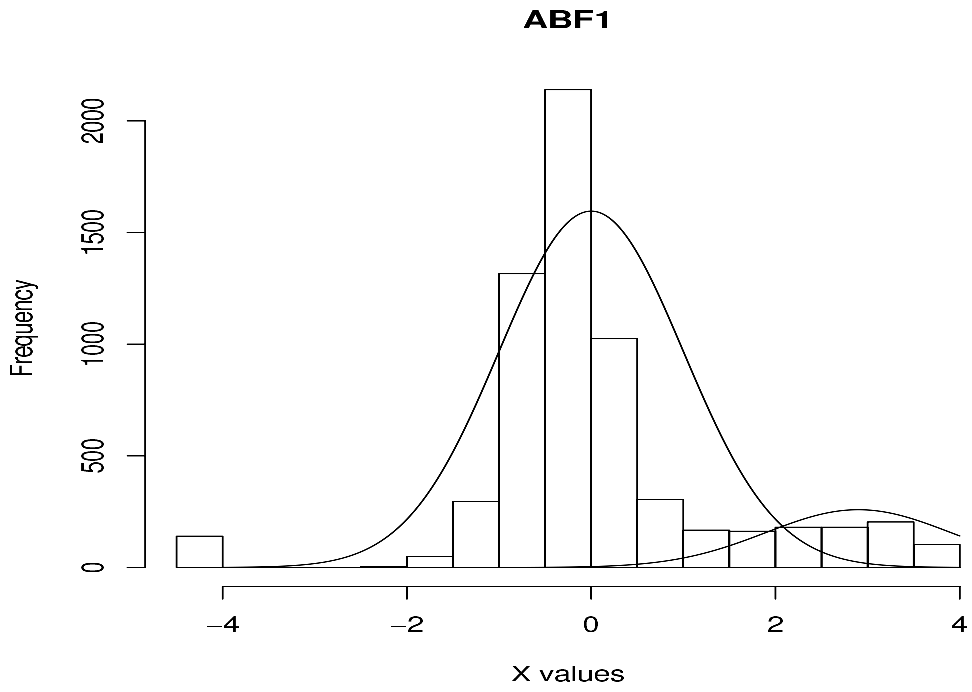

until these values converge to a fixed point. A detailed discussion of expression mixture models is given in [35]. It should be noted that our mixture model used the theoretical standard normal null distribution instead of an empirical null distribution since the use of an unrestricted mixture model (with an empirically-fitted null distribution) lead to unreasonable mixtures for several transcription factors. This procedure was repeated for each TF j to generate our full set of binding probabilities b_{ij}. The correspondence between the number of genes we predicted as enriched based on p-values ($p_i < 0.005$) and binding probabilities ($b_i > 0.5$) is very good, with a correlation of 0.97 between the number of genes predicted across our 113 transcription factors. However, we noticed that our conversion procedure tended to be overly-conservative for genes with very low p-values. In other words, genes with $p_{ij} < 0.001$ had estimated binding probabilities that where smaller than expected, possibly due to our assumption of a standard normal null distribution. For these highly-significant genes, the binding probabilities were increased to $b_{ij} = 0.95$ to reflect our extra confidence that these genes were truly enriched in the ChIP binding experiment for TF j.

8 Calculate the Probability of TF Binding Site Occurrence

The first step in calculating a probability for a binding site occurrence for TF j near to gene i is to scan the genomic sequence nearby to gene i for good matches to the position weight matrix (PWM) for TF j. The similarity between a TF PWM with width w and a sequence of nucleotides $\{a_1, \ldots, a_w\}$ is defined in the equation below:

$$S(a_1, \ldots, a_w) = \sum_t \log\left(\frac{P_{t,a_t}}{B_{a_t}}\right)$$

where P_{t,a_t} is the probability of observing nucleotide a_t in position t of the PWM, and B_{a_t} is the probability of observing nucleotide a_t in the background sequence. When scanning the sequence near gene i for matches to the PWM of TF j, we only focus on the score for the best match we find, which we denote as $S_{ij}^{\max}$.

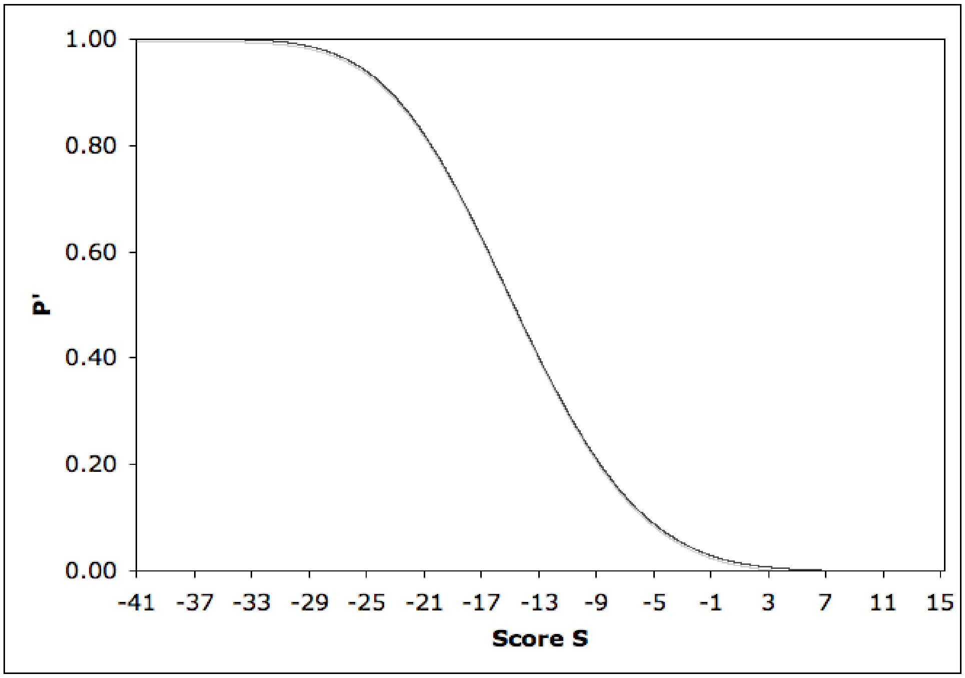

The second step of our procedure is to calculate the probability m_{ij} of a binding site for TF j near to gene i by comparing the maximum similarity score $S_{ij}^{\max}$ to the scores S that we get by applying our scanning procedure to randomly-generated w-mers (sequences of length w). More specifically, we calculate the probability of m_{ij} of a binding site for TF j near to gene i to be the probability that we observe no score S greater than $S_{ij}^{\max}$ in a randomly-generated sequence of length L, where L is the length of our original scanned sequence near gene i. In other words,

$$m_{ij} = (1 - p')^{L-w+1}$$

where $L - w + 1$ is the number of possible matches to a PWM of width w in a sequence of length L, and p' is the probability that a randomly-generated w-mer has a score S that is greater than $S_{ij}^{\max}$. As an example, for the C/EBP-β PWM, Figure shows the value of p' as a function of S.

TESS-DIST [24] was used for the calculation of these binding site probabilities. For short PWMs, this calculation can be done analytically by enumerating all possible w-mers and computing their score. However, the number of w-mers is 4^w which becomes cumbersome to enumerate as w increases so TESS-DIST adopts a dynamic programming algorithm [36] to compute the approximate distribution of scores. The approximation is achieved by binning the possible scores thus reducing the total number of possible scores to a number that is linear ($O(w)$) in the length of the PWM.

Supplementary Materials

Supplementary Table 1. Used Expression Data in Yeast

	References
1	Casagrande R, et al. (2000). Mol Cell 5 (4):729-35
2	Chu S, et al. (1998) Science 282(5389):699-705
3	DeRisi JL, et al. (1997) Science 278(5338):680-6
4	Diehn M et al.(2000) Nat Genet 25:58-62
5	Gasch AP, et al. (2000) Mol Biol Cell 11(12):4241-57
6	Gasch AP, et al. (2001).Mol Biol Cell 12(10):2987-3003
7	Gross C, et al. (2000) J Biol Chem 275(41):32310-6
8	Keller G, et al. (2001) J Biol Chem 276(42):38697-702
9	Kuhn KM, et al. (2001) Mol Cell Biol 21(3):916-27
10	Ogawa N et al.(2000) Mol Biol Cell 11:4309-21
11	Protchenko O, et al. (2001) J Biol Chem 276(52):49244-50
12	Roberts CR, et al. (2000) Science 287:873:80
13	Rutherford JC, et al. (2001) Proc Natl Acad Sci U S A 98(25):14322-7
14	Spellman PT et al.(1998) Mol Biol Cell 9:3273-97
15	Sudarsanam P et al.(2000) Proc Natl Acad Sci U S A 97:3364-9
16	Yoshimoto H, et al. (2002). J Biol Chem 277(34):31079-31088
17	Yun CW, et al. (2000) J Biol Chem 275(14):10709-15
18	Zhu G, et al. (2000) Nature 406(6791):90-4

Collected Yeast expression data from public literature and database were already processed through common microarray normalization pipelines [37]. We further normalized the combined expression profiles by subtracting the mean of each profile and dividing by the standard deviation across experiments.

Supplementary Table 2. TF pair interaction evidence

TF1	TF2	Evidence
ACE2	SWI4	Stillman et al., 1994; Dohrmann et al., 1996
ACE2	SWI5	*Doolin et al., 2001*
ACE2	SKN7	banerjee et al., 2003; Luft et al., 2001
ACE2	HSF1	banerjee et al., 2003; Luft et al., 2001
SWI4	SWI5	SGD
SWI4	SWI6	SGD
SWI4	MBP1	Dirick et al., 1996; Iyer et al., 2001; Horak et al., 2002
SWI4	STE12	Baetz et al., 2001
SWI6	MBP1	Breeden & Nasmyth, 1987; Nasmyth & Dirick, 1991; Dirick et al., 1992; Koch et al., 1993; Dirick et al., 1996; Iyer et al., 2001; Horak et al., 2002
FKH1	FKH2	Zhu et al., 2000; Hollenhorst et al., 2000; Sun et al., 2002
FKH2	NDD1	Pic et al., 2000; Koranda et al., Kumar et al., 2000; Sun et al., 2002
FKH2	MCM1	Pic et al., 2000; Koranda et al., Kumar et al., 2000; Sun et al., 2002
NDD1	MCM1	Pic et al., 2000; Koranda et al., Kumar et al., 2000; Sun et al., 2002
MCM1	STE12	Hwang-Shumm et al., 1991; Primig et al., 1991; Errede, 1993; Kirkmann-Correia et al., 1993

84 TF pairs with significant effects on expression of target genes

TF1	TF2	Gamma
ACE2	SWI4	0.200536878
ACE2	SWI5	0.22775754
ACE2	SKN7	0.394727827
ACE2	HSF1	0.432163732
SWI4	SWI5	0.200020088
SWI4	SWI6	0.35768221
SWI4	MBP1	0.213346764
SWI4	STB1	0.215075917
SWI4	FKH1	0.277864065
SWI4	FKH2	0.326079244
SWI4	NDD1	0.16672633
SWI4	MCM1	0.228995585
SWI4	ABF1	0.16068576
SWI4	CBF1	0.19898189
SWI4	RAP1	0.323818833
SWI4	REB1	0.270482368
SWI4	STE12	0.320334302
SWI5	FKH2	0.329520096
SWI5	MCM1	0.244860201
SWI5	REB1	0.307756859
SWI5	STE12	0.348633234
SWI6	MBP1	0.30583955
SWI6	STB1	0.32667736
SWI6	FKH1	0.442012906
SWI6	FKH2	0.483047343
SWI6	NDD1	0.287393258
SWI6	MCM1	0.374051579
SWI6	ABF1	0.304132418
SWI6	REB1	0.428232107
SWI6	STE12	0.480789074
MBP1	STB1	0.168895212
MBP1	FKH1	0.233766035
MBP1	FKH2	0.283392737
MBP1	NDD1	0.121526234
MBP1	MCM1	0.172403218
MBP1	ABF1	0.126488345
MBP1	MET31	0.273953855
MBP1	RAP1	0.29447829
MBP1	REB1	0.232504717
STB1	FKH2	0.351376072
STB1	NDD1	0.183789962
SKN7	HSF1	0.63765379

SKN7	YAP1	0.431132581
FKH1	FKH2	0.489508263
FKH1	NDD1	0.261916798
FKH1	MCM1	0.333256997
FKH1	ABF1	0.271478263
FKH1	RAP1	0.481893986
FKH1	REB1	0.39131322
FKH2	NDD1	0.319452542
FKH2	MCM1	0.375321032
FKH2	ABF1	0.283802259
FKH2	RAP1	0.531015871
FKH2	REB1	0.408345439
FKH2	STE12	0.467480448
NDD1	MCM1	0.20998625
NDD1	ABF1	0.13246564
NDD1	RAP1	0.293452199
NDD1	REB1	0.242339652
NDD1	STE12	0.284546001
MCM1	ABF1	0.197601091
MCM1	MET31	0.378656929
MCM1	REB1	0.335792703
MCM1	STE12	0.39270085
ABF1	BAS1	0.229871436
ABF1	CBF1	0.179409718
ABF1	GCN4	0.147673229
ABF1	GCR2	0.194112306
ABF1	HAP2	0.126568578
ABF1	HAP4	0.130782244
ABF1	MET31	0.274035349
ABF1	RAP1	0.293649063
ABF1	REB1	0.245824638
CAD1	YAP1	0.566605197
GCR2	REB1	0.379279221
HAP2	HAP3	0.285251722
HAP2	HAP4	0.241337504
HAP3	HAP4	0.336723095
HSF1	MSN4	0.534651436
LEU3	MET31	0.653046464
LEU3	RAP1	0.605344561
MET31	RAP1	0.609837895
MET31	REB1	0.482888431
RAP1	REB1	0.435230415

Supplementary Table 3. SRF Targets

Validated SRF targets		Possible False Negative
SRF		ACVR1B

NKX25
ACTR3
AOC3
RHOE
ATP2A2
CFL1
CFL2
CORO1A
DSTN
DTNA
ENAH
PDLIM5
HSPB7
ITGA5
ITGB1BP2
PFN1
PLN
SDC4
TGFB1I1
TLN1
TNNC1
TRIP6
COPEB
ELF5
ETV1
FHL2
HOXB5
LEF1
NFATC4
NFYB
TCFAP2B
WWTR1
ADM
CTGF
DUSP6
GPC4
MRGPRF
P2RX1
RRAD
TM4SF13
BIN1
DNAJB1
MRVI1
CAR3
UROD
GALNT3
IMPACT

DVL2
FEN1
NM_008273
SLC2A4
CDH11
HNRPAB
NM_010698
EEF1B2
NM_019583
NM_026307
NM_027877
IL13RA1
NDUFS1

LZF
SHKBP1

Supplementary Table 4. C/EBP-β Targets Identified by COGRIM

	Gene	Max Fold Change in ChIP-chip data	Max Fold Change in expression data
**Validated Targets Predicted by COGRIM**	Aldh1a1*	8.5	2.5
	Bcap37*	1.3	1.5
	Car3*	2.1	3.9
	Dnm2*	1.3	4.9
	Es1*	1.5	2.1
	Fkbp11*	1.8	1.7
	Gstt1*	1.3	1.6
	Grb2*	1.8	2.6
	Krt1-18*	1.2	10.7
	Pepck1*	2.8	1.4
	Rhob*	1.6	1.5
	Rnf19*	1.3	1.3
	S100a10*	3.0	2.0
	Saa1*	1.4	101.9
**Potential Targets Predicted by COGRIM**	Anxa5*	6.7	1.9
	Tcp1	4.3	1.2
	Oaz1	4.0	1.6
	Cxcl12	3.8	1.5
	Gstm1	3.3	1.8
	Ier2	3.0	1.2
	H2-Bf	2.5	1.3
	H3f3b	2.4	1.6
	Pabpc1	2.1	2.6
	Cdc42	2.1	1.3
	Arpc1b	2.1	1.6
	Aqp8	1.9	1.6
	Chd1*	1.6	1.6
	Rnase1	1.5	3.5
	Acsl1	1.5	3.9
	Ppp2cb	1.4	2.3
	Ptk2	1.3	4.2
	Rnase4	1.2	1.8

*genes also selected in Friedman et al. (2004)

Table 5. C/EBP-β Targets Identified by COGRIM based on expression data alone
*experimentally validated C/EBP-beta targets in Friedman et al. (2004)

Gene	Max Fold Change in Chip-chip data	Max Fold Change in expression data
Dnm2 *	1.30	4.90
Es1 *	1.50	2.10
Pepck1 *	2.80	1.40
Krt1-18 *	1.20	10.70
Saa1*	1.40	101.90
Ier2	3.00	1.20
Pabpc1	2.10	2.60
Ptk2	1.30	4.20
Oaz1	4.00	1.60
Prss2	1.11	2.34
Rcor1	1.25	2.06
Ifrd1	1.05	3.23
Zbtb17	1.12	11.99
Acadm	1.11	11.98
Rnf130	1.15	1.33
Tgif	1.17	2.65
Cel	1.26	1.11
Ctrl	1.32	2.56
Nme2	1.13	2.43
Cmkor1	1.27	4.60
Sort1	1.38	5.11
Coro1c	1.18	1.14
Dnajb6	1.25	5.12
Ets2	1.14	1.78
Tceal8	1.68	1.83
H2afz	1.26	2.48

Table 6. Identify Activators and Repressors
Statistical significance of the activator was determined by significant positive beta with 95% positive posterior interval, and similarly, repressor was determined by significant negative beta with 95% negative posterior interval. Sixteen TFs were identified as activators and one repressor. Also, we use another method to determine the activation or repression, and check the consistency. In the second method, statistical significance of the activator was determined by computing correlation coefficients between all TFs and all gene clusters, and taking 5% positive tail of the distribution of correlation coefficients; similarly, repressor was determined by taking 5% negative

tail. The consistent beta and expression correlation suggests that the beta parameter in our model can be used as an indicator of TFs' activity.

**TF name**	**β**	**Expression Correlation between TF and its regulons**		**Descriptions from SGD & Literature**
		**B+/C+**	**B-/C+**	
SWI5	0.35	0.36	0.53	activates transcription of cell cycle genes.
SWI6	0.63	0.63	0.67	activates transcription of cell cycle genes.
FKH1	0.57	0.77	0.75	activates transcription of cell cycle genes.
FKH2*	0.64	0.51	0.57	activates transcription of cell cycle genes.
BAS1*	0.59	0.61	0.67	Myb-related transcription factor involved in regulating basal and induced expression of genes of the purine and histidine biosynthesis pathways
CAD1	0.87	0.69	0.74	AP-1-like bZIP transcriptional activator involved in multiple stress responses, iron metabolism, and pleiotropic drug resistance; controls a set of genes involved in stabilizing proteins
HAP3*	0.46	0.41	0.56	a transcriptional activator and global regulator of respiratory gene expression
HAP4	0.41	0.84	0.80	a transcriptional activator and global regulator of respiratory gene expression
HSF1	0.89	0.55	0.59	activates multiple genes in response to heat shock
LEU3*	0.81	0.60	0.64	involved in branched chain amino acid biosynthesis and ammonia assimilation
MET31*	0.72	0.55	0.56	involved in regulating expression of the methionine biosynthetic genes
MSN4	0.56	0.59	0.64	Transcriptional activator related to Msn2p; activated in stress conditions, which results in translocation from the cytoplasm to the nucleus; binds DNA at stress response elements of responsive genes, inducing gene expression
RAP1*	0.75	0.55	0.58	DNA-binding protein involved in either activation or repression of transcription

REB1*	0.58	0.56	0.57	RNA polymerase I enhancer binding protein
STE12	0.69	0.83	0.84	activates genes involved in mating or pseudohyphal/invasive growth pathways
YAP1	0.50	0.58	0.54	bZip transcription factor required for oxidative stress tolerance
RME1*	-0.45	-0.51	-0.46	Zinc finger protein involved in control of meiosis; prevents meiosis by repressing IME1 expression

* COGRIM identified seven more activators as well as one repressor RME1 when comparing to GRAM [4].

Supplementary Materials

Supplementary Figure 1.
Based on our estimated regulation indicators C_{ij}, we construct the functional regulatory network with 2298 TF-gene interactions between 39 TFs and 1542 functional target genes. The edges represent the regulatory activity of TF to target genes, the box nodes (red) represent transcription factors labeled with TF names, other nodes represent the target genes and the gene names are not shown. The regulatory network is illustrated with LGL [38].

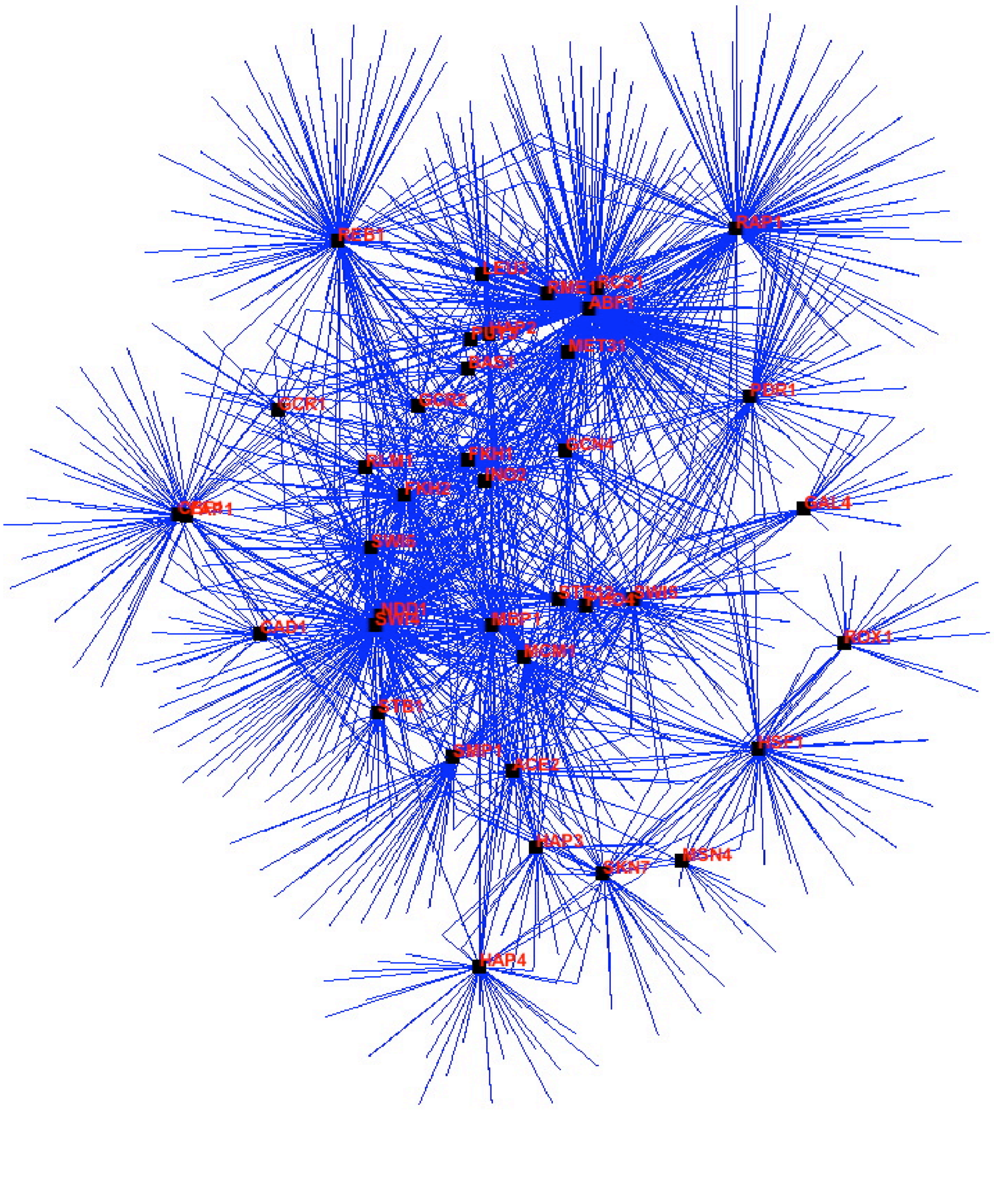

Supplement: Additional data file 1 — Detailed model procedures, Gibbs sampling algorithm, data processing procedures, predicted gene targets, and annotation evidence. [file gb-2007-8-1-r4-S1.pdf]
